# Incorporating networks in a probabilistic graphical model to find drivers for complex human diseases

**DOI:** 10.1371/journal.pcbi.1005580

**Published:** 2017-10-12

**Authors:** Aziz M. Mezlini, Anna Goldenberg

**Affiliations:** 1 Department of Computer Science, University of Toronto, Toronto, Ontario, Canada; 2 Genetics and Genome Biology, The Hospital for Sick Children, Toronto, Ontario, Canada; University of California, San Diego, UNITED STATES

## Abstract

Discovering genetic mechanisms driving complex diseases is a hard problem. Existing methods often lack power to identify the set of responsible genes. Protein-protein interaction networks have been shown to boost power when detecting gene-disease associations. We introduce a Bayesian framework, Conflux, to find disease associated genes from exome sequencing data using networks as a prior. There are two main advantages to using networks within a probabilistic graphical model. First, networks are noisy and incomplete, a substantial impediment to gene discovery. Incorporating networks into the structure of a probabilistic models for gene inference has less impact on the solution than relying on the noisy network structure directly. Second, using a Bayesian framework we can keep track of the uncertainty of each gene being associated with the phenotype rather than returning a fixed list of genes. We first show that using networks clearly improves gene detection compared to individual gene testing. We then show consistently improved performance of Conflux compared to the state-of-the-art diffusion network-based method Hotnet2 and a variety of other network and variant aggregation methods, using randomly generated and literature-reported gene sets. We test Hotnet2 and Conflux on several network configurations to reveal biases and patterns of false positives and false negatives in each case. Our experiments show that our novel Bayesian framework Conflux incorporates many of the advantages of the current state-of-the-art methods, while offering more flexibility and improved power in many gene-disease association scenarios.

## Author summary

Networks and pathway-based methods are commonly used to improve the power of gene detection in associations with complex human diseases. Network diffusion approaches have shown their effectiveness and superior performance in cancer studies. Still, there are many problems such as noise and missingness with currently available human networks that bias the results of gene detection. We propose a novel graphical model-based method Conflux that overcomes several of the pitfalls of the existing state-of-the-art approaches while building on their successes. Conflux integrates genotype data with networks directly, using diffusion-like methods, but only as part of a structure in a probabilistic model to reduce the negative effect of the noise in the networks. This Bayesian framework allows Conflux to keep track of the uncertainty in the gene list that is being associated with the disease and consequently rank the genes with respect to our confidence in the association. It also allows for the discovery of gene sets that are not fully supported by the network if they have enough support in the data. These improvements result in a flexible approach that improves the power in many gene-disease association scenarios while reducing the number of false positives reported.

This is a *PLOS Computational Biology* Methods paper.

## Introduction

Identifying genes associated with complex human diseases is the holy grail of human genetics. Years of research have proven this task to be very difficult, requiring substantial data and methodological resources, and far from being solved [1–4]. Recently, protein-protein interaction (PPI) networks have been successfully used to improve the power of detecting genes associated with genetic diseases [5]. Examples of success stories include Crohn’s disease and diabetes [6], autism and related disorders [7, 8], multiple cancers [9] and many others [10, 11]. Some of the first methods developed to take advantage of gene and protein interaction networks, such as dmGWAS, DAPPLE, NIMMI, iPINBPA, jActiveModules [12–16], directly relied on the network annotations to iteratively search for neighbourhoods of associated genes while greedily exploring the network. More recently, several methods have used network diffusion to identify subnetworks associated with disease. Diffusion methods take into account the local topology of the network (degrees of nodes, average random walk distance) in order to propagate the heat (association signal) and then identify hot subnetworks. Hotnet and Hotnet2 [17, 18] are examples of network diffusion methods successfully used to discover modules associated with several cancers. Although Hotnet2 was initially applied on gene-based statistics from cancer data, several recent publications used it successfully in Genome Wide Association Study (GWAS) context discovering subnetworks of genes statistically associated with diseases such as Ulcerative Colitis and ADHD [19].

The drawback of the previous methods is their reliance on the marginal statistics of each gene, such as p-values of marginal gene significance, to diffuse over the network. Recent methods [20, 21] go beyond the diffusion of test statistics by ‘smoothing’ or propagating the data from each individual patient, i.e mutations, across the network. In this type of network propagation approach, genes neighbouring the gene with a mutation get some of its signal even if they are not mutated in the considered patient. For example, NBS [20] used this approach to identify relevant cancer subtypes and then selected subnetworks that had strong associations with these subtypes. In [21], the approach was used to identify silent genes that are neighbours of mutated or differentially expressed genes. While this postulates an interesting hypothesis for how complex diseases arise, it is very hard to test such a hypothesis in a computational setting, as essentially all the evidence for the importance of these genes stems from the network with no direct support from the patients’ genetic data. More recently, [22] proposed an ILP algorithm for detecting subnetworks that are mutated in a large fraction of patients. This approach offers many advantages over methods using gene summary statistics: i) it directly optimizes a function of interest (explaining the most patients); ii) it takes advantage of underlying structures in the data such as mutual exclusivity between genes, such that solutions that include genes that are mutated in different groups of patients when taken together are considered preferable to sets of genes that are all mutated in the same patient. This type of structure was not directly exploited in methods such as [20, 21] that used gene-based test statistics for diffusion. NBS [20], ILP [22] and the silent-gene [21] methods mentioned above were successfully applied to TCGA cancer data. Unfortunately, this idea is not directly applicable to heritable diseases because there are many more germline genetic variants in cases and controls compared to thousands of somatic mutations specific to cancer. Additionally, none of the methods mentioned above account for the uncertainty in predicting which genes are associated with the phenotype, given the limited sample size.

In this paper, we propose a biologically motivated hierarchical graphical model to identify sets of genes explaining complex human diseases from exome data. Probabilistic graphical models (PGMs) [23] are a framework to represent joint distributions and the conditional dependencies among variables. Hierarchical PGMs were previously used in computational biology, for example to identify regulatory network modules [24] and infer haplotype blocks [25]. Here we define a new PGM model to effectively address the problem of disease mechanism identification while incorporating PPI network knowledge into the framework.

Similarly to Hotnet2, our method is flexible enough to work on both cancer and hereditary diseases, and similarly to [22], our method uses the full genetic data, not just summary statistics, taking into account complementarity and mutual exclusivity between genes to find the set of genes that explains the most patients. Most network-based approaches directly rely on the structure of the network when searching for gene subnetworks of interest. They either use the PPI network as a guide and directly select subnetworks from it, or they use it to diffuse information across genes. In addition to incorporating the characteristics of the current state-of-the-art methods, encoding the network as part of the structure of a probabilistic graphical model allows our method to be less susceptible to the noise intrinsic to protein-protein interaction networks. Our approach is able to find relevant sets of genes even when the sets are not or only partially supported by the network but are supported by the DNA aberrations in and across patients, limiting the influence of incompleteness and noise in the networks. Finally, our method returns marginal probabilities for each gene rather than a fixed associated subnetwork, keeping track of the uncertainty in predicting gene-disease associations.

We tested our approach to identify a set of genes driving a complex human disease from exome data in a wide variety of settings. We first sampled various network structures comparing our probabilistic method to a very widely used diffusion method, Hotnet2, as well as the most widely used rare-variant aggregation method SKAT-O [26]. We then compared the ability of both methods along with other network methods including dmGWAS, JActiveModule and PINBPA as well as enrichment method DAVID [27], to recover true sets of genes previously identified in epilepsy, schizophrenia, autism and ovarian cancer from the simulated exome data. Our experiments indicate that our approach has higher sensitivity and precision than Hotnet2 in > 90% of the considered scenarios. We conclude that not relying on the network structure directly but encoding it as part of a structure in a Bayesian graphical model is a powerful new way to improve gene driver discovery in complex human diseases.

## Results

To date there is no complex disease for which the set of gene drivers is fully known and thus it would be impossible to assess false positive rate to properly compare the novel and existing methods considered in this study on real rare variant data. We thus simulated complex diseases arising in a population due to rare variants using the standard European population model with the optimal parameters as in [28]. To simulate causal genes in random experiments, we picked sets of 10, 20 or 50 genes (depending on the desired disease complexity) from local neighbourhoods in iRefIndex Network [29]. In real disease cases, we used the published sets of genes for four complex diseases (see real disease section). To generate disease in the population that is caused by the selected gene set, we sampled the top 3,000 individuals in the population, proportionally to the mutation burden in the causal genes. From this diseased population we then sampled 100, 200 or 400 cases. We randomly sampled matching number of controls from the healthy population. These case-control scenarios, generated based on realistic population simulations and observed associations in real diseases, provide a robust framework to compare methods on scenarios where PPI networks can potentially help to identify disease mechanisms.

### Performance on simulated complex diseases

**Fig 1:**
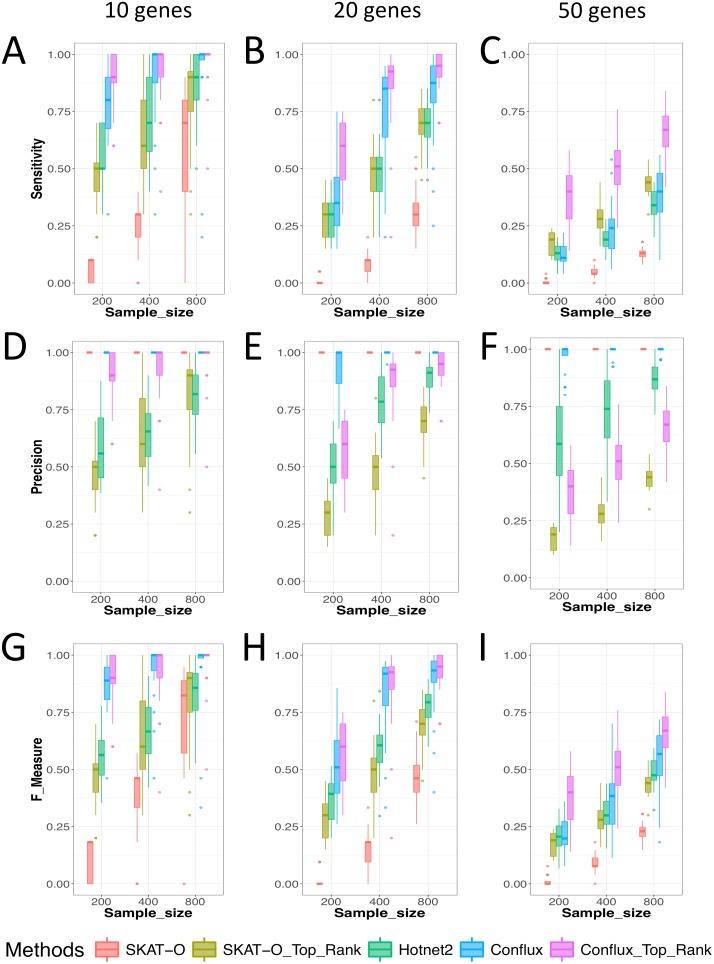
Sensitivity (A-C), precision (D-F) and F-Measure (G-I) as a function of the complexity of the disease (the number of selected causal genes), evaluated for our method (Conflux), SKAT-O and Hotnet2. The columns represent increasing complexity of simulated diseases: 10, 20 and 50 causal genes respectively, from left to right. The sample size is varied from 200 to 800 on the x-axis of each experiment (the sample size is equal to the number of cases plus the number of controls). Sensitivity (Power) is the proportion of simulated causal genes that are detected by the method. Precision is the proportion of detected genes that are true causal genes as opposed to false positives. The F-Measure is a harmonic mean of sensitivity and precision.

We assessed the performance of SKAT-O [26], Hotnet2 applied on gene p-values from SKAT-O, and our graphical-model-based approach Conflux. Similarly to most other network-based methods, the output of Hotnet2 is a fixed set of genes (subnetwork) that is associated as a whole with the disease studied. A gene is either included in this list or not. In contrast, Conflux returns marginal probabilities for each gene. SKAT-O returns p-values for every gene. Thus, both Conflux and SKAT-O allow us to examine prioritization and ranking of genes. For this reason, we evaluate SKAT-O and Conflux in two ways: i) selecting genes based on thresholds, SKAT-O p-values (< 4.10^−6^) and Conflux marginal probabilities (≥ 0.2), the values chosen to control for Type I error; ii) selecting the top *P* ranked genes, where *P* is the number of causal genes. We assessed sensitivity, precision and the F-Measure (a harmonic mean of precision and sensitivity) for each considered method. Fig 1 presents the results across all simulations, each bar in the barplot summarizing 20 simulated disease scenarios.

Fig 1A–1C show that Conflux has significantly higher sensitivity in detecting the causal genes compared to Hotnet2 in both thresholding and ranking regimes in 88% of scenarios. Fig 1D–1F show that Conflux has a consistently lower rate of Type 1 errors than Hotnet2. We attribute these observations to the fact that Hotnet2 and many other diffusion methods focus on identifying sets of genes that form significant subnetworks together, not paying as much attention to the false positives that might also be part of that subnetwork. Conflux has two advantages over those methods: i) it tries to maximize the number of patients explained while minimizing the number of selected genes; ii) it keeps track of the uncertainty of each of the genes thus the gene might not be selected even if it is well connected within the subnetwork. Across all choices of sample sizes and number of causal genes, Conflux top ranked genes is the best performing approach in terms of power (sensitivity) and F-Measure, followed closely by Conflux with thresholded marginals (≥ 0.2). Both significantly outperform Hotnet2 in all scenarios in terms of F-Measure. This is mostly due to Hotnet2 reporting more false positives.

Analyzing the performance of SKAT-O’s top ranking genes, we can see that SKAT-O ranks causal genes high even if they do not reach significance, evident from the comparison of SKAT-O thresholded approach (in red) with SKAT-O top ranked genes (in olive). Interestingly, as the complexity of the disease grows (20 and 50 genes), the sensitivity of Hotnet2 is the same or even lower than SKAT-O top ranked genes. At the same time, the precision of Hotnet2 is much higher than that of SKAT-O top-ranked genes. We conclude that Hotnet2 does a great job at selecting the true causal genes among SKAT-O top ranked and significant genes (taken as input), which is helped by the knowledge encoded in the network structure.

Top ranking versions of both SKAT-O and Conflux have higher F-Measure and sensitivity compared to the thresholding versions of the same methods across all scenarios. This means that i) significance thresholding keeps both methods on the conservative side and ii) it is beneficial to examine gene rankings and not just fixed gene sets. Both versions of Conflux perform better than the best (gene ranked) version of SKAT-O indicating that independently of the complexity of the disease and the number of available samples, Conflux has higher accuracy in prioritizing causal genes than the most widely used non-network gene prioritization method.

As expected, we see that the problem of identifying the causal genes is more difficult for more complex diseases, i.e. the performance of all methods goes down for 20 and 50 causal diseases (second and third columns of Fig 1). The power/sensitivity of all methods and variations increased with larger sample sizes. Hotnet2’s precision increased from an average of 50% at *n* = 200 to an average of 90% at *n* = 800. It is the only non-ranking method exhibiting this behaviour. This can be explained by the fact that it is constructing a subnetwork from SKAT-O top-ranked genes (SKAT-O’s precision is affected by sample size) and maximizes the significance of the whole subnetwork rather than its constituent genes independently.

To summarize, our results on randomly simulated causal disease genes show that both network-based methods, Conflux and Hotnet2, outperform SKAT-O, which does not use networks, in all of our simulations, indicating the importance of using networks in identifying interacting disease associated genes. Additionally, Conflux outperforms Hotnet2 according to the F-measure in all scenarios, which signifies the importance of using the full data in combination with the network rather than diffusing gene summary statistics.

### Performance on local network configurations

Local network topology plays an important role in how the information gets diffused across the network. When propagating messages/heat between genes, Conflux limits the incoming signal received by a gene from its neighbours, while Hotnet2 limits the heat coming out of a particular gene. This means that both methods penalize highly connected nodes but in different ways. We thus decided to investigate specific configurations of local network topologies and assess method performance in each of these scenarios. We examined three patterns: star, clique and chain and selected causal gene sets accordingly. We applied our method to the whole network, i.e. all genes, preserving the patterns of interest as significant, and examined genes identified by each of the methods.

#### Star-shaped subnetwork

**Fig 2:**
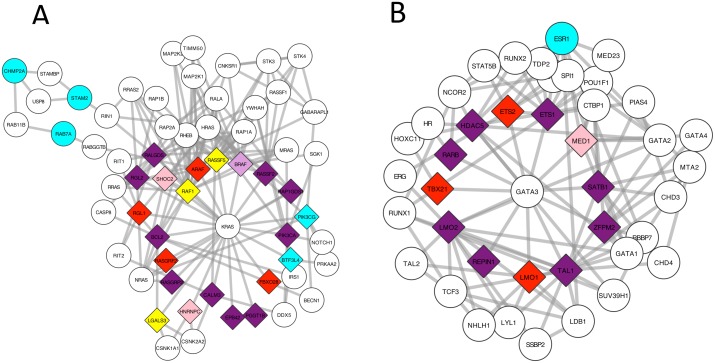
Results of Hotnet2 and Conflux on two Star-shaped disease subnetwork where the center is not a causal gene. A) *KRAS* centered star B) *GATA3* centered star. The nodes in purple are genes found by both Hotnet2 and Conflux. The nodes in cyan were only found by Hotnet2. The nodes in red and pink are respectively nodes detected (marginal ≥ 0.2) or having suggestive evidence (marginal ≥ 0.05) by Conflux. Nodes colored in plum were found by Hotnet2 but only have suggestive evidence in Conflux. Yellow nodes are true causal genes that were neither found nor suggested by any method. The diamond shaped nodes are the true causal genes. The sample size used is *n* = 800.

The first configuration is a star shaped subnetwork where peripheral genes are causal but the center is not. This setting explores how much each method is driven by the network connectivity alone and tests whether some genes would be called positives just because of their local neighbourhood. In complex diseases, our goal is to find genes that have some evidence of harmful variants in the patients. Fig 2 shows the performance of Conflux and Hotnet2 on two such configurations: 1) *KRAS* centered; 2) *GATA3* centered, genes annotated as involved in many cancers and other complex diseases. In both scenarios, both methods detected a majority of the true genes and did not identify the center of the star as causal. Conflux is specifically designed to guarantee that no gene would be called positive based on its neighbourhood alone by limiting the influence of the network, encoded as the prior. Hotnet2 does not explicitly limit the incoming messages and performed well due to the combination of heat scores and an influence matrix which prevents nodes with low heat scores from having high influence.

Among the 22 true causal genes around *KRAS*, 10 were found by both methods, 4 were only found by Conflux and 3 were only found by Hotnet2, i.e. the sensitivity of Conflux and Hotnet2 are 63% and 59% respectively. An additional 3 genes were highly ranked by Conflux (marginal above 0.05 but below 0.2) one of which was found by Hotnet2 and two that were not found by any method. Hotnet2 reported 3 false positives bringing its precision to 81%.

Among the 12 causal genes around *GATA3*, 8 were found by both methods and 3 were only found by Conflux. Thus the sensitivity of Conflux and Hotnet2 are 91% and 66% respectively in this scenario. The only gene that was not found by either method was highly ranked by Conflux (marginal above 0.05 but below 0.2). Hotnet2 reported one false positive gene bringing its precision down to 88%.

#### Clique-shaped subnetwork

**Fig 3:**
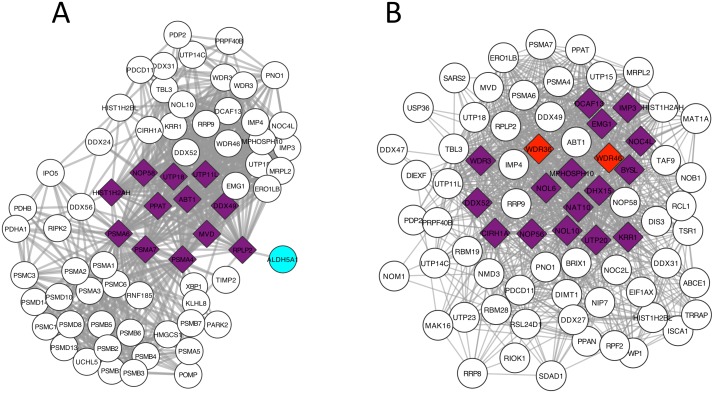
Results of Hotnet2 and Conflux on clique-shaped disease subnetworks where the disease genes are part of an even larger clique. (A) Causal subnetwork contains a third of the genes in the clique; (B) Causal subnetwork is half of the genes in the largest clique. The nodes in purple are genes found by both Hotnet2 and Conflux. The nodes in cyan were only found by Hotnet2. The nodes in red and pink are respectively nodes detected (marginal ≥ 0.2) or having suggestive evidence (marginal ≥ 0.05) by Conflux. Nodes colored in plum were found by Hotnet2 but only have suggestive evidence in Conflux. The diamond shaped nodes are the true causal genes. The sample size used is *n* = 800.

In this experiment we took the largest Clique (fully connected subnetwork) from the iRefIndex network and we selected i) a third (Fig 3A) and ii) a half (Fig 3B) of its genes randomly as the causal genes. In this scenario, each true gene is acting as the center of a star connecting to all other genes in the clique. As opposed to the previous example, here the selected centres are indeed causal and most of peripheral genes are not. We know from previous literature that Hotnet (and to a lesser extent Hotnet2) can have false positives generated by “hot” star centers. We expected that these hot star centers would diffuse their heat to all the genes in the clique, generating a large number of false positives. Fig 3 shows that both Conflux and Hotnet2 performed well in this setting.

In the case of a third of the clique (12 genes) being causal (Fig 3), both methods were able to recover all the genes. Against our expectations, Hotnet2 reported only one false positive gene and it was not part of the clique. In the second case (Fig 3B), where half of the clique (18 genes) were selected as causal, 16 were found by both methods and the remaining two were found only by Conflux. The sensitivity for Conflux is 100% and for Hotnet2 is 88%. For reference, SKAT-O detected 58% and 38% of the causal genes in these respective scenarios.

We investigated further why Hotnet2 did not return a large number of false positives. We noticed that Hotnet2 heat diffusion step did not perceptibly affect the final results especially for genes with zero or very low initial heat scores (log p-values). The reason is the large difference of scale between the heat scores (usually between 6 and 30) and the influence matrix coefficients (most of non-diagonal values being less than 0.01 especially for nodes with 10 interactions or more). This means that the heat exchanged between nodes is so low that if a node is cold it never becomes hot because of its neighbours. We verified that the false positives of Hotnet2 are highly ranked non-significant genes by SKAT-O (valid for the star configuration as well).

#### Chain-shaped subnetwork

Finally, we simulated chain-shaped disease subnetworks. To generate the set of genes in a chain-shaped subnetwork we start from a seed gene and we incrementally select genes as follows:

- The newly selected gene is in the direct neighbourhood of the last selected gene.
- The selected gene does not have direct connections to any of the previously selected genes except the last (reduces looping).
- We favour genes with the least number of connections
- At least 2 connections are needed to make a chain.

**Fig 4:**
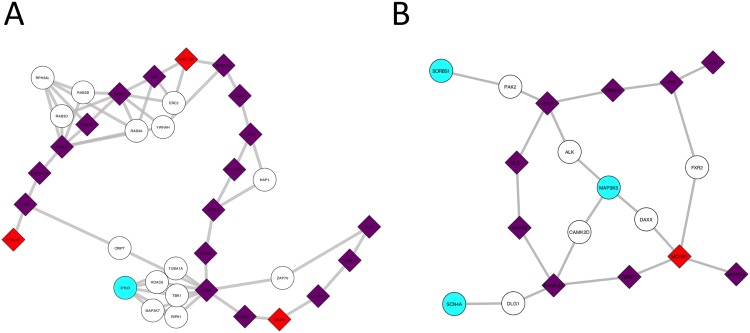
Results of Hotnet2 and Conflux on two radomly generated chain-shaped disease subnetworks. (A) Chain of 20 causal genes. (B) Chain of 10 causal genes. The nodes in purple are genes found by both Hotnet2 and Conflux. The nodes in cyan were only found by Hotnet2. The nodes in red and pink are respectively nodes detected (marginal ≥ 0.2) or having suggestive evidence (marginal ≥ 0.05) by Conflux. Nodes colored in plum were found by Hotnet2 but only have suggestive evidence in Conflux. The diamond shaped nodes are the true causal genes. The sample size used is *n* = 800.

Since Hotnet2 uses an influence matrix summarizing all distances in the network while taking into account local topology [18], we expect it to be favoured over Conflux which only uses up to second order neighbourhoods (the direct neighbours and their neighbours) and ignores more distant genes. In a chain with relatively few branching connections, many genes are expected to be relatively close together in the influence matrix while Conflux will consider at most four genes in the chain at a time (two before and two after the current gene). Fig 4 shows that Conflux is doing better in this scenario. In both chains, it uncovers all causal genes and has no false positives: Hotnet2 had one false positive and three false negatives on Fig 4A, three false positives and one false negative on Fig 4B. We attribute this to the fact that Conflux uses the full data to find the set of genes that can explain the most patients thus gaining power over Hotnet2 that uses summary statistics (SKAT-O p-values). Overall, Conflux had 100% sensitivity in both scenario while Hotnet2 had 85% and 90% for chain A and B respectively. In comparison, the sensitivity of SKAT-O is 40% and 80%.

### Performance on real disease gene sets

**Fig 5:**
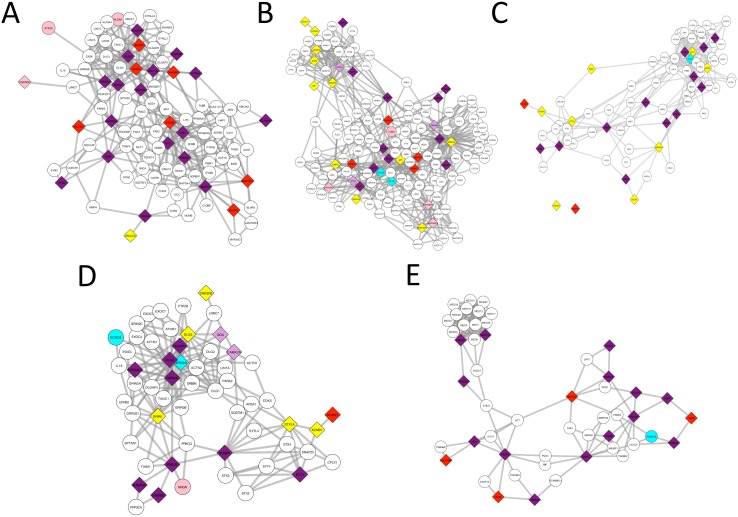
Results of Hotnet2 and Conflux on a literature reported disease subnetwork. (A) Schizophrenia. (B) Epilepsy. (C) ASD1. (D) ASD2. (E) Ovarian Cancer. The nodes in purple are genes found by both Hotnet2 and Conflux. The nodes in cyan were only found by Hotnet2. The nodes in red and pink are respectively nodes detected (marginal ≥ 0.2) or having suggestive evidence (marginal ≥ 0.05) by Conflux. Nodes colored in plum were found by Hotnet2 but only have suggestive evidence in Conflux. Yellow nodes are true causal genes that were neither found nor suggested by any method. The diamond shaped nodes are the true causal genes. The sample size used is *n* = 800.

In all the simulations above, the disease causing genes were selected based on explicit assumptions such as taking them from local neighbourhoods in section on simulated complex diseases or local network configurations above. In this section, we avoid making such assumptions by taking real disease-associated gene sets reported in the literature. The exome data is still simulated the same way as in the previous sections. We assessed the performance of network-based methods on several gene sets associated with autism spectrum disorder (ASD), epilepsy, schizophrenia [8] and ovarian cancer [30] in the literature. Each of these gene sets are subnetworks that can be found in iRefIndex network and were not originally found by Hotnet or Hotnet2 to be fair to all methods considered. We ran our experiments simulating a set of 400 patients and 400 controls. The results are summarized in Fig 5.

#### Schizophrenia

The reported subnetwork associated with schizophrenia contains 26 genes and is illustrated in Fig 5A. Out of the 26 genes, Conflux detected 24 as causal while Hotnet2 applied on SKAT-O p-values detected 17. This means 95% and 67% power respectively for Conflux and Hotnet2. In comparison, SKAT-O detected 30% of the genes. Additionally, one more true gene was highly ranked by Conflux although with a lower marginal probability.

#### Epilepsy

The reported subnetwork associated with epilepsy contains 35 genes and is illustrated in Fig 5B. Out of the 35 genes, 11 were found by both methods and 14 were not found by either of them. For the remaining genes, 5 were only found by Hotnet2 and 4 were only found by Conflux. This means 42% and 45% power respectively for Conflux and Hotnet2. However, Conflux ranked 6 other true genes highly: 3 of the genes only found by Hotnet2 and 3 of the genes not found by either method. If these genes were considered it would bring the power of Conflux to 60%. Neither method reported false positives. In this case, having marginals for each gene and having the ability to rank them allows Conflux to consider more relevant genes and potentially improve the accuracy of gene detection. In comparison, SKAT-O detected just 17% of the correct genes.

#### ASD 1

The reported ASD-ID module illustrated in Fig 5C has 23 genes, 12 of them were found by both methods and 8 were not found by either of them. Of the remaining 3 genes, 2 were only found by Conflux and 1 was only found by Hotnet2. The power is 60% for Conflux and 56% for Hotnet2. In comparison SKAT-O detected 39% of the genes. It is interesting to point out that one of the true genes uniquely found by Conflux, *KCNMA1*, is actually not in the neighbourhood of the other causal genes in the iRefIndex network. This shows that since Conflux is only using the network as a prior, it can still identify genes even if they are not necessarily connected to others in the considered network. Hotnet2, on the other hand, is unable to find one isolated gene but we have seen examples in our simulations where it found a group of 2 or 3 genes that are not close to the rest and added them as a separate component, showing Hotnet2’s bias to the evidence of connectivity in the network.

#### ASD 2

The reported ASD with ID module has 18 genes and is illustrated in Fig 5D. 9 of them were found by both methods and 5 were not found by either of them. Of the remaining 4 genes, 3 were only found by Hotnet2, with two of them being highly ranked by Conflux (marginal above 0.05), and 1 was only found by Conflux. The power is 55% for Conflux and 66% for Hotnet2. There were no false positives reported by Conflux while Hotnet2 reported one false positive gene. If we consider the genes found by Conflux with marginals above 0.05 then both methods have equal performance. In comparison SKAT-O detected 44% of the genes.

The performance of Conflux for this particular disease subnetwork is lower than what we reported for similar disease complexity when we considered random simulations above. By looking at the data we found two reasons for this behavior: 1) A good proportion of the signal in the patients was contained in three genes: *STXBP1*, *PRKCB*, *GABRG2*. These genes were easy to detect and their marginal probabilities were equal to 1. This left a larger tail of genes that were very rarely mutated in our patients and much more difficult to find especially for a conservative methods such as Conflux that avoids reporting potential false positives; 2) The true causal genes were spread around the PPI network and most of them only had one or two neighbours even when we consider second order interactions (direct neighbours and their neighbours). This limited the ability of Conflux to take advantage of the network, an interesting finding that can be certainly improved in the prior directly.

#### Ovarian cancer

The reported subnetwork associated with ovarian cancer contains 18 genes and is illustrated in Fig 5E. Conflux detected all of the 18 genes while Hotnet2 applied on SKAT-O p-values detected 14. This means 100% and 77% sensitivity (power) for Conflux and Hotnet2 respectively. In comparison, SKAT-O detected only 44% of the genes indicating that the high performance of both network methods is not due to the simulated scenario being too easy. Using the network information is crucial to improve the power of gene-disease associations. Conflux did not report any false positives and Hotnet2 reported one.

The results on the literature reported gene sets are consistent with our observations for the randomly generated disease subnetworks. For the sample size used (*n* = 800), the precision of Hotnet2 is similar to what we previously found (around 90%). In almost all scenarios we observe more false positives for Hotnet2 than for Conflux. Conflux has similar or higher sensitivity than Hotnet2 in most scenarios especially if we consider the top-ranked genes.

Cytoscape’s jActiveModules plugin [16], PINBPA [15] and dmGWAS [12] are also network based methods that search for a set of genes, i.e a subnetwork associated with the disease. These methods adopt an iterative greedy search strategy within the network to find significant modules. We ran all three methods on the five disease gene sets experiments (for sample size equal to 800) and found much lower performance than the diffusion-based Hotnet2 and our method Conflux. Details and results can be found in the Section 4.1 in S1 Text. Additionally, we ran pathway based analysis using DAVID [27] to illustrate the difference with network based methods and designed a logistic regression baseline (Results in Sections 4.2 and 4.3 of S1 Text).

## Discussion

Inspired by the successes of network-based methods such as Hotnet2 in finding novel gene-disease associations, we propose a novel alternative for using PPI networks. We present a graphical model based framework where a PPI network is incorporated into the structure of the probabilistic model to empower the discovery of genes associated with complex diseases. Our method, called Conflux, performs better than Hotnet2, SKAT-O, dmGWAS, jActiveModule, PINBPA, DAVID and a logistic regression-based variant aggregation method in a variety of gene-disease association scenarios. Both Conflux and Hotnet2 methods outperformed marginal gene testing with SKAT-O, indicating that information propagation over the network are likely to improve the performance in gene-disease association studies.

Conflux has several advantageous features that explain its improved performance. First, it uses the genotype data directly rather than gene-based test statistics which means it takes advantage of hidden structure in the data (such as mutual exclusivity between genes) in order to find the set of genes that explain the most patients. Second, it uses the PPI network as part of the probabilistic model which means that it can find associated gene sets even if they are not part of a strong subnetwork in the PPI network provided to the method. And third, it offers guarantees about the influence of the PPI network and limits the amount of false positives that are due to noise inherent in all currently available protein-protein interaction networks in humans, as is illustrated in our robustness examples in Section 3 in S1 Text. There are of course disadvantages to probabilistic frameworks, for example, compared to diffusion methods such as Hotnet2, it has more parameters and takes longer to do the inference. The beauty of the Bayesian setting is that even with more parameters, we can apply our approaches to relatively few samples without being concerned for overfitting: if there is not enough evidence in the data, the genes will not be selected into the disease mechanism, i.e. their likelihood of being associated with the phenotype will remain very small, close to the prior.

Additionally, as a Bayesian method Conflux keeps track of the uncertainty in associating each individual gene to the disease. Thus, instead of returning fixed gene sets (which is the case for most network-based methods such as Hotnet2), it outputs the marginal probabilities associated with each individual gene. This more informative output provides an opportunity to perform gene prioritization and ranking. Furthermore, we compute the marginal probabilities for each variable associated with a gene and/or patient which helps us to prioritize which genes and variants are more likely to cause the disease in a given patient. This information can further be used to make clinical decisions specific to the patient to maximize the effect of a treatment, for example. This is an advantage of a Bayesian framework over methods reporting scores or ranks or fixed gene lists for the population.

In our experiments, we use the iRefIndex network. It is one of the networks that were previously processed and used by Hotnet2 with a defined hyper-parameter beta proposed for their method. We also tried the Biogrid and HumanNet networks with no observed change in Conflux performance. The choice of network should not affect our results as long as it provides evidence for the underlying disease mechanism. If the given network does not contain any evidence for the interaction between causal genes, our method is likely to perform similarly to SKAT-O, whereas other network based methods would not be as applicable.

We observed that Hotnet2 performed well at optimizing gene selection given a set of non-zero heat scored genes when genes were close together in the network. However, we also observed that the heat diffusion process itself did not have a perceptible impact on that performance as the weights in the influence matrix were too small to make a difference. Varying the choice of scales for the influence matrix and heat scores could help to improve Hotnet2 performance even further.

We ran a variety of simulations to assess the performance of diffusion methods, represented by Hotnet2, and Conflux in different scenarios. Some of the biases observed are a propensity for Hotnet2 subnetworks to include false positives genes, especially when in a lower sample size setting, and a tendency to miss out on isolated true genes that are not close to the rest of the true genes in the network. For Conflux, we observed a lower power when all of the true genes are close but not tightly interconnected. This is due to the fact that Conflux only considers network neighbours of first and second order (direct neighbours and their neighbours) and can be improved upon if we modify it to take a matrix representing the full continuum of distances within the network similarly to the influence matrices used by Hotnet2. However, doing so will require the selection of a hyper-parameter similar to the *δ* parameter in Hotnet2. Currently, Hotnet2 returns four sets of results corresponding to four choices of delta and there is no clear way of automatically selecting the right *δ* in a real disease scenario. In our experiments we took the best performing result of Hotnet2 when the ground truth was known. Using cross validation where we withhold part of the data samples, might be a solution for both methods to select the right hyper-parameter.

Despite Conflux and Hotnet2 methods having higher power to detect gene-disease associations compared to other network-based methods, enrichment methods and standard methods that do not use networks, there is still room for improvement. Our results, show that we are still underpowered for highly complex diseases (larger number of genes) and for diseases where the causal genes are not close together in the network. Some of the possibilities for further improving our power to detect disease-associated genes is to make use of harmfulness predictions as priors over the genetic variants considered (prior over the *X* variables). There are many approaches for estimating the harmfulness predictions, especially for coding variants and those estimations can easily be integrated into the prior of our Bayesian framework in the same way we integrated PPI networks. That would offer an advantage over the current approaches that either pre-select variants based on harmfulness or transform harmfulness predictions into weights in numerous ways. Using them as priors is less restrictive since Conflux would learn which variants are important from the data even when the knowledge encoded in the prior is noisy and incomplete. Similarly, our framework can easily incorporate prior knowledge over genes (*H* variables) in order to encourage genes that are specific to a tissue of interest or genes that were reported as associated with the phenotype in previous papers or by other methods.

### Conclusions

It is becoming increasingly clear that networks are a powerful tool in performing gene-disease associations, improving the power of gene discovery. There are many ways in which networks could be incorporated into the analysis. The existing diffusion methods are effective, yet are subject to a few limitations. Our proposed Bayesian framework, Conflux, addresses limitations such as dealing with network noise and combining networks directly with the variant information rather than relying on precomputed summary statistics. It also proposes additional advantages, such as providing the ability to rank genes using marginal probability estimates. As researchers strive to integrate more information into a joint model of genomic data in an attempt to discover novel gene-disease associations, our powerful Bayesian framework is bringing flexibility and power to the modeling paradigm.

## Methods

### Conflux graphical model

**Fig 6:**
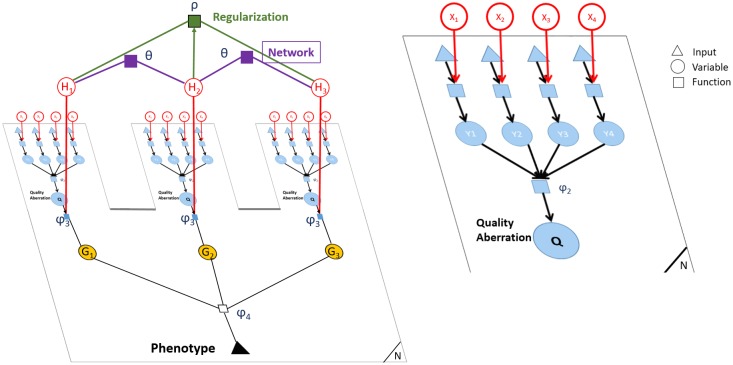
Conflux’s hierarchical graphical model. Graphical model representing the relation between phenotypes, coding variants and gene latent variables with the PPI network used as prior. All the variables, factors and inputs inside the plate are per individual. The variables, factors and inputs outside the plate, such as protein-protein interactions are not individual-specific. This model simultaneously uses all genes genome-wide and is shown here for 3 genes for clarity. The graph on the right is a zoom in on the gene specific portion of the graphical model.

Conflux jointly models the outcome (disease/no disease), the genotypes and the PPI network in a hierarchical graphical model. The graphical model is designed to fit the hierarchical structure of our problem by describing all variants in the data, all genes and all individuals (cases/controls). The hierarchical structure aggregates variants into gene variables and gene variables into the phenotype. Fig 6 shows a factor graph corresponding to our model. We use variables specific to each individual and indicator variables that are shared across individuals. The variables X, D and Y correspond to the variants. The variables H, Q and G correspond to the genes. X and H are indicator variables for the variants and genes respectively, while D and Q are patient specific variables for the variants and genes respectively. Y and G are intermediate variables that are also patient specific variables for variants and genes, they represent the product of D and Q with the corresponding indicator variables (X and H respectively). Table 1 describes all the listed variables.

**Table 1:** Description of the variables in the model. *pheno* and *D* are fixed inputs. All other variables are latent random variables to be inferred.

Variable	Description
*i*	Index used for variants.
*j*	Index used for individuals (cases/controls).
*g*	Index used for genes.
*D*_*ij*_	Genotype of coding variants *i* in a patient *j*. *D* is the exome data.
*X*_*i*_	Indicates whether variant *i* affects the function of the protein.
*Y*_*ij*_	*X*_*i*_ ⋅ *D*_*ij*_. Indicates whether coding variant *i* is affecting individual *j*.
*Q*_*gj*_	Aggregates the functional variants present in individual *j* and gene *g*.
	*Q*_*gj*_ takes on values {0, 1, 2} indicating a normally functioning, a partially (e.g. haploinsufficiency) or fully dysfunctional protein.
*H*_*g*_	Indicates whether the gene g is associated with the disease.
	The posteriors over *H*_*g*_ are the main output of Conflux.
*G*_*gj*_	*Q*_*gj*_ ⋅ *H*_*g*_ Indicates if the gene *g* is relevant to the phenotype in patient *j*.
*pheno*_*j*_	Phenotype of the individual *j* (case or control).

The PPI network is encoded as factors (priors) over the **H** variables (Θ). For every interaction in the PPI network, we add a factor linking the pair of genes involved. Additionally, a regularization factor *ρ* is added over **H**, to encourage a set of disease-associated genes to be sparse. This factor is implemented in the form of cardinality potentials [31].

The likelihood of the model can be written as:

$$\begin{array}{ccc} P(X,G,H,Q,pheno|D) & \propto & P\left(pheno|G\right)P\left(G|H,Q\right)P\left(Q|X,D_{exome}\right)P\left(X\right)P\left(H\right) \end{array}$$ (1)

where the priors are defined as follows: *P*(*X*) are non-informative (set to a constant value 0.5), *P*(*H*) = *θ* ⋅ *τ* ⋅ *ρ*. There are two types of priors used for the *H* variables:

1. The network prior is encoded in the factors Θ connecting each pair of genes known to interact from existing protein-protein interaction networks. These factors make two interacting genes more likely to be both relevant to a given phenotype as opposed to a random pair of genes that do not interact. It also helps to bring interacting genes into the set of phenotypically relevant genes if one or both of them are not marginally significant. We use asymmetric directed factors parametrized by the degree of the target gene in the network to account for the large discrepancy in degree distributions for different genes (see section on PPI network implementation below). Conflux considers both first order and second order interactions (neighbours of neighbours).
2. The sparsity prior is encoded in two ways: a) a constant small prior *τ* over all genes indicating that it is unlikely for any random gene to be involved in the phenotype; b) a regularization factor *ρ*, mentioned above, encouraging only a few *H*_*i*_ are active at once.

The conditional probabilities are defined on the graph as the *φ* factors. The probability distribution of *Q* depends on the state of the intermediary variables **Y**. The relationship is described by the factor *φ*_2_(*Q*, **Y**) = *P*(*Q* | **Y**) = *P*(*Q*|*X*, *D*). For more details, see Section 2.1 of S1 Text.

For a particular gene and individual, the *G* variable depends on *Q* and *H* as described by the factor: $\varphi_{3}\left(G,H,Q\right)=1_{\left[G=H\cdot Q\right]}$. This factor encodes that a gene can only be relevant in a particular individual if the gene is shown to be relevant to the phenotype in general and if that particular individual has potentially harmful exome variants in that gene.

Finally, the phenotype (disease/no disease) is given as an input. It relates to the number of active *G* variables in each patient: a patient is likely to have some affected genes while a healthy individual should have few or no affected genes. The expected number of affected genes for patients and the tolerated number of affected genes in healthy population depend on the disease and its complexity and are therefore taken as parameters in the function *φ*_4_(*pheno*, *G*) = *P*(*pheno*|*G*) by our method. (More on *φ*_4_ in Section 2.2 in S1 Text).

### Conflux inference

For inference, we use loopy belief propagation, modified for efficiency (see below), to jointly infer the marginal distributions of the unobserved variables in our graphical model. After convergence of the loopy belief propagation our method returns estimates of the posterior marginal probabilities for the *H* and *G* variables, along with the ranked list of most relevant aberrations (coding variants) in each affected individual according to the model.

All computed messages are normalized and kept in log space for numeric stability. We used message damping with parameter *α* = 0.5 to improve the convergence behaviour of the algorithm. While there is no theoretical guarantee of convergence, we find that in practice dampening of the messages leads to convergence in every case we have considered.

Variables related to the aberrations in each gene and patient are the most numerous, and their messages are the most expensive to compute and the slowest to vary. Conversely, the messages between the phenotype, the *G* and *H* variables are the fastest to change since these variables are tightly correlated. Therefore, every time we update the messages for *X*, *Y* and *Q* variables, we update the messages between *G*, *H* and phenotype variables up to 10 times or until their local convergence.

Some of the factors connect a large number of nodes to one node: the *φ*_4_ factor connecting all *G* variables to phenotype, the regularization factor *ρ* connecting the *H* variables together, and the *φ*_2_ factor connecting *Y* nodes to a *Q* node. Since each of these factors mainly depends on the sum of its input variables, the computation becomes feasible by using the approach in Tarlow et al [31], in which a binary tree structure is used, with the internal nodes representing the intermediary sums of variables, and then belief messages are computed across the tree. This divide & conquer approach reduces the complexity of messages computation to a *O*(*nlog*^2^(*n*)) where *n* is the number of initial variables considered.

### Conflux implementation of a PPI network as a prior

The PPI network is used as prior over the *H* variables indicating whether a gene is relevant to the disease. It is encoded by the Θ factors, one for every pair of interacting genes. Θ encourage pairs of interacting genes to be active together for the same disease. There are difficulties associated with the implementation of this prior:

- The connectivity degree is very different across genes. While some genes can be connected to tens or even hundreds of neighbours, other genes have very few or no connections. This can create large biases pushing some genes to be always spuriously associated with the phenotype or pushing genuinely important genes down.
- We want to encourage interacting genes to be relevant together in the same disease, but we do not want to penalize genes when their neighbours are not related to the disease. An edge in the PPI network means the two genes involved could potentially have similar function in relation to disease. But a gene involved in the disease can still have many interactions with genes that are not part of the disease mechanism.
- PPI networks are considered very noisy and incomplete with a large proportion of true interactions missing from the network. It is also possible that genes have indirect interactions with each other which makes them relevant to the same disease.

To avoid having our network prior biasing Conflux results, we take the following measures. First, instead of the symmetric indirected factors usually used for modeling joint probabilities, we make the Θ factors into directed and asymmetric factors. The idea is that we want to encourage a gene to be ‘on’ if its neighbour has some evidence of being associated with the disease, but we do not want to encourage a gene to be ‘off’ because its neighbours are off. Consequently, if the neighbor is off it should be non-informative of the state of the considered gene. The directed factor between a gene *i* and its neighbour *j* is therefore: *P*(*H*_*i*_ = 1|*H*_*j*_ = 0) = 0.5, *P*(*H*_*i*_ = 0|*H*_*j*_ = 0) = 0.5, *P*(*H*_*i*_ = 1|*H*_*j*_ = 1) = *δ*, *P*(*H*_*i*_ = 0|*H*_*j*_ = 1) = 1 − *δ*. This is very similar to the heat diffusion in Hotnet2 which is also asymmetric, and only positive.

Second, we parametrize the directed factor by the degree of the target gene. The goal is that the accumulation of signals from a large number of neighbours should never be enough to make a highly connected gene seem disease associated. So the parameter *δ* from the factor definition is a function of the degree of gene *i* in the network. *δ* is chosen to limit the amount of influence this gene can receive from any one of its neighbours. In Hotnet2, the weights in the influence matrix also depend on the nodes degrees, but they depend on the degree of the source node (the influencer) while our *δ* depends on the degree of the receiving node. This approach allowed us to reduce the number of false positives across many of the presented examples.

To compute *δ* for a particular degree *d*, we first compute the maximal contribution of the network to the posterior. In Conflux, every gene starts from a small prior *τ* and needs evidence from its own data (coding variants) to reach large enough marginals to be considered a contributor to the disease in question. The maximal possible contribution *M*_*c*_ is the contribution that would make the gene’s marginal reach 0.02 (starting from the initial prior *τ* and supposing the gene have no signal). Limiting ourselves to this maximal contribution from the network guarantees that the network context on its own will never associate the gene with the disease (marginal of at least 0.2 needed) on its own. Messages between binary probabilistic variables are represented by the ratio of the probability of being active and the probability of being inactive. The maximal contribution *M*_*c*_ is given by:

$$\begin{array}{c} \frac{\tau }{1-\tau }\;\cdot \;\frac{M_{c}}{1-M_{c}}=\frac{0.02}{1-0.02} \end{array}$$ (2)

Once we define the maximal contribution, we compute the value of *δ* insuring that the sum of messages coming from a neighbourhood that is significantly enriched in active genes will be equal to the maximal contribution. Any neighbourhood that is less than significant would make less than the maximal contribution. A neighbourhood significance is assessed by a binomial with the probability of success in a single trial is equal to the prior *τ* and the number of trials is equal to the degree *d*. The significance threshold used is 0.05 divided by the total number of genes. For example, if the prior *τ* is 0.0025 and the degree *d* is 10, significance is obtained if there are *a*_*min*_ = 3 active neighbours and *δ* is chosen so that the sum of messages (in log space) coming from 3 active nodes (parametrized by delta) would be equal to the maximal contribution. In non-log space, the product by *a*_*min*_ becomes a power, this gives:

$$\begin{array}{c} {\left(\frac{\delta }{1-\delta }\right)}^{a_{min}}=\frac{M_{c}}{1-M_{c}} \end{array}$$ (3)

For the same prior *τ* = 0.0025 but with a higher degree *d* = 100, significance is only obtained if there are *a*_*min*_ = 6 active neighbours and *δ* is chosen so that the sum of messages coming from 6 active nodes would be equal to the maximal contribution. Therefore, higher degree nodes require more active neighbour genes to reach significance so they will have a lower *δ*. Additionally, we ensure that delta is never larger than 0.9 to limit the influence of one gene on another even if they are the sole neighbour. Note that the examples above are computed for a specific set of parameters and are given merely as illustration of the method.

Up to this point our computations are theoretical, i.e. before the inference process and without looking at any real messages. It is possible that during the inference we observe more active neighbours than the minimal significant number *a*_*min*_ we utilized to compute *δ* which would generate higher total contributions than the maximal contribution. Therefore, we also cap the sum of contributions to the maximal contribution during the inference process.

### Overview of the simulation process

To simulate a disease, we selected *P* genes [P is 10, 20 or 50] that are close to each other on the protein-protein interaction network as being the causal disease mechanism. To do so, we randomly selected a seed gene in a iRefIndex Network [29], then randomly picked other causal genes from first and second degree neighbourhoods. The first and second degree neighbourhoods around the seed gene must jointly have an acceptable size, i.e larger than the number of causal genes being simulated. Although these simulation assumptions (causal genes being in relatively close proximity in the network) may not always reflect the (unknown) reality of how real disease genes are distributed in PPI networks, they still provide a realistic enough framework to assess scenarios where PPI networks should be able to help to identify a disease mechanism. Note that in addition to these simulations we also used other configurations of causal genes (star, clique and chain) and literature reported disease subnetworks (see the Results).

For simulating coding variants, we used the European population model with the optimal parameters as in [28]. We considered the single nucleotide variants (SNVs) with selection coefficient (indicating impact on the fitness) greater than s = 0.001 as the deleterious SNVs and the remaining SNVs as neutral, as recommended in [32]. The generated sequence including the random missense SNVs was randomly split into blocks, each block corresponding to one of the genes in the iRefIndex network. Using this approach we generate coding variants (the vast majority of which being rare variants) in ≈ 12,000 genes for 900,000 individuals.

For every individual in the simulated population, we count the number of harmful mutations within the disease mechanism and we select the patients as the top 3,000 individuals according to that sum. The rest of the population is considered healthy. We thus generated our case/control setting with sample size *n* for analyzing the disease by selecting $\frac{n}{2}$ cases from the patient subpopulation and $\frac{n}{2}$ controls from the healthy subpopulation.

### Alternative methods: SKAT-O and Hotnet2

SKAT-O is the most widely used statistical test for the association of rare variants with a phenotype [26]. It combines a burden test with a variance component test (SKAT [33]) and has been proven to do well in terms of power and type 1 error. SKAT-O is resilient to the presence of neutral and protective variants. In our simulations, all the deleterious variants with high selection coefficient were rare variants. Therefore a gene-based test for aggregating rare variants such as SKAT-O is the appropriate baseline.

Hotnet2 [18] takes gene-based statistics as an input. Though originally developed and used for cancer data, Hotnet2 was previously used in a GWAS setting identifying genes associated with complex human diseases through common variants [19]. In our example for complex human diseases due to rare rather than common variants, we used SKAT-O’s p-values (after negative log transformation) as input to Hotnet2. As recommended in [19] we only selected the top scoring genes to have non-zero scores because Hotnet2 does not do well when there are many genes with low heat scores.

We tested multiple ways of thresholding the p-values to improve the performance of Hotnet2. First, we attempted to use FDR q-values and only selected those genes with FDR<0.05. This created two problems: i) Hotnet2 does not run with a limited number of genes (less than 20) satisfying this FDR threshold; ii) the performance of Hotnet2 dropped considerably in terms of sensitivity and became closer to that of thresholded SKAT-O. This is due to the fact that SKAT-O is underpowered and restricting the analysis to only those genes detected by SKAT-O limits the potential of Hotnet2.

We also attempted to use the local FDR curves [34] and look for inflection points as suggested in [19]. But in most examples this approach gave much worse performance for Hotnet2 and was very unstable from one simulation to the next: Hotnet2 was either too conservative (low sensitivity) or too imprecise (precision under 0.1). We chose to select the genes with p-values below a relaxed FDR criterion (<0.5). If the number of genes passing that criterion was less than 20, which was the case for most of our experiments, we augmented it by taking genes with the smallest 20 p-values to ensure Hotnet2 can run. We also attempted taking the top 50 and top 100 genes, but that had a negative effect on Hotnet2 performance where the precision dropped to below 50%.

Hotnet2 returns four sets of results corresponding to four different estimations of the method’s hyper-parameter *δ*. Given that there is no way to select the right set of results we always reported the best performing set returned by Hotnet2. This might give an unfair advantage to Hotnet2, but we did not find a more principled way of automatically selecting the right delta hyper-parameter for Hotnet2.

### Literature-reported subnetworks

We extracted disease associated subnetwork from two publications. Hormozdiari [8] describes a combinatorial optimization method MAGI for building disease associated modules. From their supplementary data we selected 4 modules: Schizophrenia_M2, Epilepsy_M1, ASD_ID_M2 (referred to as ASD1 in this paper) and ASD_with_ID_M2 (referred to as ASD2). [30] construct informative gene subnetworks by integrating cancer gene expression profile with a PPI network and is applied on Ovarian cancer. We selected the module they report in their paper.

## Supporting information

S1 Text Supplementary details about the method. (PDF)
