# Supplementary material for: Incorporating networks in a probabilistic graphical model to find drivers for complex human diseases
Source: PLoS Comput Biol. 2017 Oct 12;13(10):e1005580. doi: 10.1371/journal.pcbi.1005580 (PMC5638204; doi:10.1371/journal.pcbi.1005580)
Supplement: S1 Text — (PDF) [file pcbi.1005580.s001.pdf]

Supplementary information

Aziz M. Mezlini

April 30, 2017

1 Belief Propagation Algorithm

The formulas for the messages' computations are generated following the conventional approach defining two types of messages: factors to nodes and nodes to factors.

The message μ going from a factor to a node is the marginalization over all the other nodes of the product of all incoming messages η by the factor itself. The message η going from a node to a factor is equal to the product of all the incoming μ messages to that node from all other neighbour factors. Note that the priors are also factors. For example, having a prior h over an H variable is the same as sending a message $(1-h, h)$ to the corresponding H variable.

The order chosen for updating the messages helped with the speed of the algorithm and the convergence. We further improved the convergence behaviour of the algorithm by damping messages which help avoiding oscillatory behaviours. Every time we update a message μ the new value becomes

$$\mu_{updated} = (1-\alpha)\mu_{old} + \alpha\mu_{new} \tag{1}$$

, where α is the damping parameter (by default we take $\alpha = 0.5$).

2 Graphical model

2.1 Factor φ_2

For a gene g and an individual j, Q_{gj}, aggregates the functional variants present in that individual and gene (part of Y_j variables) into one latent variable. Q_{gj} takes on values $\{0, 1, 2\}$ indicating a normally functioning, a partially dysfunctional (e.g. haploinsufficiency) or a fully dysfunctional/inactivated protein The probability distribution of Q depends on the state of the $\mathbf{Y}$ variables attached to it and the relationship is described by the factor $\varphi_2(Q, \mathbf{Y}) = P(Q \mid \mathbf{Y})$. In our implementation we use the φ_2 factor in table 1:

In other words, if the individual have no functional coding variants at all in the considered gene (i.e. $Y_i = 0$, $\forall i$), we have Q is necessarily 0 (No disruption in the considered gene and individual). If there is an i such that $Y_i = 2$ (i.e. the individual have at least one homozygous functional variant in the considered

$Q\|\mathbf{Y}$	$\exists i$ such that $Y_i = 2$	$\exists i$ such that $Y_i = 1$, and $\forall i$ $Y_i < 2$	$Y_i = 0$, $\forall i$
0	0	0	1
1	0	$0.5^{(-1+\sum Y)}$	0
2	1	$1 - 0.5^{(-1+\sum Y)}$	0

Table 1: φ_2 factor or the conditional distribution of Q given Y

gene), then we have Q is necessarily 2 (both alleles are compromised). In the absence of homozygous functional variants, the probability distribution of Q also depends on the number of heterozygous variants and their repartition across alleles. We assume the haplotypes are not phased. In that case, if there is an i such that $Y_i = 1$ (i.e. the individual have heterozygous functional variants in the considered gene), we have the probability of $Q = 1$ (the gene is only partially dysfunctional) is the probability of all variants falling into the same allele. Therefore,

$$\varphi_1(Q = 0.5, \mathbf{Y}) = P(Q = 1 \mid \mathbf{Y}) = 0.5^{(-1+\sum Y)} \tag{2}$$

2.2 Factor φ_4

The factor φ_4 encodes the relation between the number of active G variables in each individual and the phenotype (disease/no disease). A patient is likely to have some affected genes while a healthy individual should have few or no affected genes.

The expected number of affected genes for patients and the tolerated number of affected genes in healthy population depend on the disease and its complexity. Therefore, φ_4 is parametrized to reflect that. There are two parameters to φ_4 . They encode the probability of being a control given that the individual have a certain number of disrupted disease-associated genes (sum of G variables). First, a fixed parameter c_0 indicating the probability of being a control (healthy individual) if the individual has no disrupted disease-associated genes (The sum of G is 0). This probability depends on how many of the cases/patients will have an observable disruption in the data analyzed. For example, in some diseases we can expect only a small proportion of patients to have causal disruptions that are contained in their exome data. Therefore an individual with no disrupted disease-associated genes can either be a control or a case with the causal variants unobserved. c_0 is selected by cross validation. Second, we added an additional free parameter that control the probabilities of being a control given a strictly positive number of disrupted disease-associated genes (sum of G variables). c_d is a decay parameter. $c_d = 0.05$ for example, means that an individual has only a 0.05 probability of being a control if they have one disrupted disease-associated gene and $(0.05)^n$ probability of being a control if they have n disrupted disease-associated genes, for $n > 1$. In our current implementation c_d is added to the graphical model as an additional probabilistic variable that can takes values in (0.05 , 0.1, 0.2, 0.4) and we put a uniform prior on its distribution if we do not have a prior knowledge about the disease complexity. It is inferred with all the

other variables with the loopy belief propagation framework. c_d can also be fixed to one value selected by cross-validation.

2.3 Sparsity priors

Our two sparsity priors τ and ρ depend on the expected number of causal genes m in the disease considered. τ is a constant small prior indicating that any particular gene have a small chance a priori to be associated with the disease. It is equal to the ratio of m over the total number of genes. ρ is a regularization prior encoded as a Gaussian with mean m and standard deviation of $m/2$. The Gaussian is truncated to be positive. In theory m can be a hyper-parameter selected by cross-validation. However, we observed that performance was not significantly affected if we change m within an order of magnitude (See Section 3.4). In all our experiments, $m = 20$ was chosen and it did as well as giving the right number of causal genes.

3 Robustness analysis

3.1 In depth analysis of performance

Conflux combines multiple elements together to improve the power detect disease genes: Variants aggregation within a hierarchical graphical model and PPI network incorporation as a prior. To show the individual contributions of both these elements, we run experiments where we remove the factors encoding the network prior altogether from our framework. Figure 1 shows that ignoring the network can negatively affect the performance of Conflux. For example, the sensitivity is reduced by 26% if we ignore the network when the sample size is 400. This is equivalent to detecting 3 less causal genes on average. We observe a similar drop in performance when we limit the network prior to the direct interactions instead of looking at second order neighbours. This is expected since in the simulations, causal genes are sampled from a second order neighbourhood and rarely directly interact with each other. Nevertheless, Conflux is still doing better than gene-based testing methods such as SKAT-O and the burden test CAST, even without using the network. This is due to the fact that we do not test each gene individually but instead search for a set of genes that, together, explain the most patients. Such a joint analysis of all genes (as opposed to univariately testing each gene) could also be performed by a regression-type approach on all genes/variants with the phenotype as a response variable. As a baseline, We implemented a logistic regression with a group lasso regularization penalty to encourage variants within the same gene to be taken or ignored together. Unfortunately, this approach was over-parametrized and did not perform well in our simulations even with high regularization coefficients (See Section 4.3).

3.2 Robustness to network noise

We also tested the effect of perturbing the network by randomly removing edges or adding spurious edges. Figure 2 shows that such perturbations do not have a significant effect on Conflux performance. This robustness to network noise is an important feature of Conflux because it uses the PPI network as a prior rather than considering it a ground truth and looking for submodules within the network itself.

3.3 Effect of changing the marginals' threshold

Methods such as Hotnet2 and dmGWAS return a fixed list of associated genes as an output. In order to compare fairly with these methods, we decided to take a threshold over the genes marginal probabilities and consider any gene above the threshold as a positive. This way, all methods now have comparable outputs: a fixed set of genes, and we can assess the performance by looking at the Sensitivity and Precision of each method. In the main paper, we picked a threshold of 0.2 for all experiments because in practice it keeps a tight control on type-1-error even under the null hypothesis (no causal genes). Table 2 shows how the results would change if we pick a different value for the threshold. The results are shown for the real genes sets experiments with sample size 800.

Table 2: **Effect of changing the marginals' threshold on Conflux 's performance**

	**Sensitivity**				**Precision**			
**Threshold**	**0.5**	**0.2**	**0.1**	**0.05**	**0.5**	**0.2**	**0.1**	**0.05**
Schizophrenia	0.88	0.92	0.92	0.96	1	1	1	0.92
Epilepsy	0.34	0.43	0.54	0.6	1	1	1	0.95
ASD1	0.61	0.61	0.61	0.61	1	1	1	1
ASD2	0.5	0.55	0.61	0.67	1	1	1	0.92
Ovarian cancer	1	1	1	1	1	1	1	1

As we can see, the choice of threshold does not strongly affect the performance of Conflux in terms of sensitivity and precision. Most true causal genes have high enough marginals and are well separated from other genes having low marginals. For the Epilepsy and ASD2 experiments, sensitivity continue to increase as we reduce the threshold beyond 0.2 without a cost to the precision. This indicates that our choice of threshold equal to 0.2 is on the conservative side.

3.4 Robustness to the Tau hyperparameter

Tau is a small prior on the genes indicator variables H. It encodes that any one gene has a priori a small chance of being associated with the disease since we assume that only a small proportion of all genes will be involved in the disease mechanism. Figure 3 shows that Conflux 's performance does not change much for different values of tau. Since we are simulating 20 genes, the correct Tau

prior should be 20 divided by the total number of genes ($m = 20$). We observe that taking any Tau within an order of magnitude does not have a large effect on performance, with smaller Tau values being slightly more favorable in the lower sample size settings.

4 Comparing to other approaches

4.1 JActiveModules, PINBPA and dmGWAS

JActiveModules and PINBPA are two plugins of Cytoscape for finding associated subnetworks within a network. We loaded the iRefIndex network and the p-values from SKAT-O into Cytoscape and ran both methods using the default parameters. We considered the real genes sets experiments with sample size 800. For each of the five disease genes sets, JActiveModules highest scoring module contained more than half of all genes (≥ 7000). Therefore, the performance was always very low because of the low precision. We examined the five highest scoring modules for every experiment and they never contain more than two causal genes. PINBPA did slightly better. The best returned module contained between 745 and 1135 genes depending on the disease and contained most of the causal genes. However, the precision is still very low with such a large number of false positives, and the performance is much lower than that of Conflux or Hotnet2. The only exception is the Epilepsy experiment where PINBPA returned a module of only 25 genes 19 of which were causal resulting in a sensitivity of 0.54 and a precision of 0.76. The F-measure 0.63 is therefore on par with that of Conflux and Hotnet2 (≈ 0.60) on that experiment. Table 3 shows the performance of PINBPA best scoring module. We also investigated the top 5 modules but the subsequent modules still contained a large number of genes but had less less than 2 or 3 causal genes each.

Table 3: **Performance of PINBPA. Best scoring module**

	**Sensitivity**	**Precision**	**F-Measure**	**Conflux F-Measure**
Schizophrenia	0.62	0.017	0.033	0.96
Epilepsy	0.54	0.76	0.63	0.6
ASD1	0.65	0.02	0.039	0.75
ASD2	0.77	0.016	0.031	0.71
Ovarian cancer	0.94	0.015	0.029	1

We also ran dmGWAS on all five disease experiments. Table 4 shows that dmGWAS is working reasonably well but the performance is still lower than that of Conflux or Hotnet2.

4.2 Gene set enrichment analysis

Unlike network methods, gene set enrichment analysis test a predefined fixed set of genes for association with the phenotype. The gene set is usually defined

Table 4: **Performance of dmGWAS in finding the causal genes**

	**Sensitivity**	**Precision**	**F-Measure**	**Conflux F-Measure**
Schizophrenia	0.27	0.87	0.41	0.96
Epilepsy	0.14	1	0.24	0.6
ASD1	0.26	0.85	0.4	0.75
ASD2	0.39	1	0.56	0.71
Ovarian cancer	0.22	0.8	0.34	1

by a biological process, function or pathway. If the genes set is overrepresented in the top ranked genes by association to the phenotype (ranked by p-values for example), the whole set is considered "enriched". In our experiments, the causal genes are selected based on the PPI network iRefIndex. Therefore, it is unfair to compare to gene set enrichment method given that the causal genes might not be together in one of the predefined gene sets tested. Knowing that there is some overlap between PPI networks and predefined gene sets such as pathways, we decided to run a gene set enrichment analysis method (DAVID) on some of our experiments for purely illustrative purposes (We are aware the comparison is not fair). David takes a set of genes as input(no ranking). We ran DAVID on the genes with nominally significant p-values from the SKAT-O test. We considered all 5 disease experiments and we fixed the sample size to 800.

DAVID returns a list of enriched gene sets (pathways for example). If we were to consider the full pathway returned, the precision of this approach will be very low as only a small proportion of the genes in the pathway are responsible for the enrichment. The second alternative is to only report those genes that made the pathway enriched. Both approaches have the same sensitivity, therefore we will adopt the second approach since it has a better precision.

On the schizophrenia experiment, DAVID returned only one enriched gene set: Postsynaptic density with an FDR of 0.015. The set contains 184 genes but only 12 genes were responsible for it being enriched: 8 true causal genes and 4 false positives.

In the Epilepsy experiment , DAVID returned four overlapping gene sets: GOTERM-CC-direct synaptic vesicle ($FDR = 510^{-5}$), synaptic vesicle docking ($FDR = 510^{-5}$), synaptic vesicle cycle ($FDR = 510^{-5}$) and GOTERM-BP-direct neuro-transmitter secretion ($FDR = 0.03$). The four sets respectively contain 92,8,63,51 genes and only 11,5,9,7 genes were respectively responsible for the enrichment. Taking the union of these genes gives 12 true causal genes and 3 false positives.

In the ASD1 experiment DAVID returned the GOTERM-CC-Direct cell junction gene set ($FDR = 0.001$). The gene set contain 459 genes only 19 of which contributed to the enrichment. Of these 9 were true causal genes and 10 were false positives.

In the ASD2 experiment, DAVID returned 3 overlapping genes sets: Post-synaptic membrane ($FDR = 410^{-3}$), amphetamine addiction ($FDR = 910^{-3}$) and cocaine addiction ($FDR = 0.01$). The gene sets contain 211,66 and 49 genes

respectively . If we consider only the genes responsible for the enrichment we have a total of 11 true causal genes and 8 false positives.

In the Ovarian cancer experiment, DAVID found no enriched gene set.

If we consider only the genes responsible for the enrichment and not the whole returned gene set, the performance is summarized in Table 5

Table 5: **Performance of DAVID in finding the causal genes**

	**Sensitivity**	**Precision**	**F-Measure**	**Conflux F-Measure**
Schizophrenia	0.3	0.66	0.41	0.96
Epilepsy	0.34	0.8	0.48	0.6
ASD1	0.39	0.47	0.42	0.75
ASD2	0.61	0.58	0.59	0.71
Ovarian cancer	0	0	0	1

The sensitivity being lower than that of network-based methods is due to the fact that pathways and predefined gene sets do not overlap the set of all causal genes very well. The relation between causal genes is better described in the network in our simulations.

4.3 Baseline Regression Approach

As a baseline approach for associating genes and variants with a phenotype, we implemented a logistic regression with group lasso regularization. Here, group lasso is used to aggregate rare variants per gene. The logistic regression is ran taking all the exome variants genome wide as predictors. The group lasso penalty encourages variants within the same gene to be taken or ignored together, allowing in a way for the aggregation of the variants within the same gene. We also added interaction terms corresponding to the edges in the PPI network (iRefIndex). The interaction term is the product of the indicator variables indicating whether a given gene is mutated. From the features selected by the regression, we estimate how many are related to one of the causal genes and how many are not and then we use these numbers to compute the sensitivity, precision and F-measure similarly to what we did to assess the performance of Conflux and Hotnet2. For the choice of regularization term lambda, we selected the value that gives the best performance for this approach (in F-measure). Therefore, the results reflect the best possible performance that could be obtained by this approach. We ran this approach on all 5 real gene sets experiments. The results are summarized in Table 6

5 Individual-specific posteriors

Given the model at convergence and the variants an individual have, we can compute the probability of the individual being affected by the phenotype. We did this for the 5 disease experiments after fixing the sample size to 800. The results are shown in Figure 4.

Table 6: **Performance of the logistic regression baseline in finding the causal genes**

	**Sensitivity**	**Precision**	**F-Measure**	**N-variables**
Schizophrenia	0.11	0.25	0.15	44
Epilepsy	0.17	0.42	0.24	74
ASD1	0.34	0.61	0.44	76
ASD2	0.27	1	0.43	44
Ovarian cancer	0.05	0.08	0.06	48

Figure 4 show that our model does well in separating the cases (red) from the controls (black). A few healthy individuals are wrongly predicted as cases because they have variants in disease associated genes, and there is not enough evidence to consider the variant as neutral (very rare variant or singleton). The affected individuals that do not have high probabilities are affected through genes that did not have enough signal to be found by Conflux.

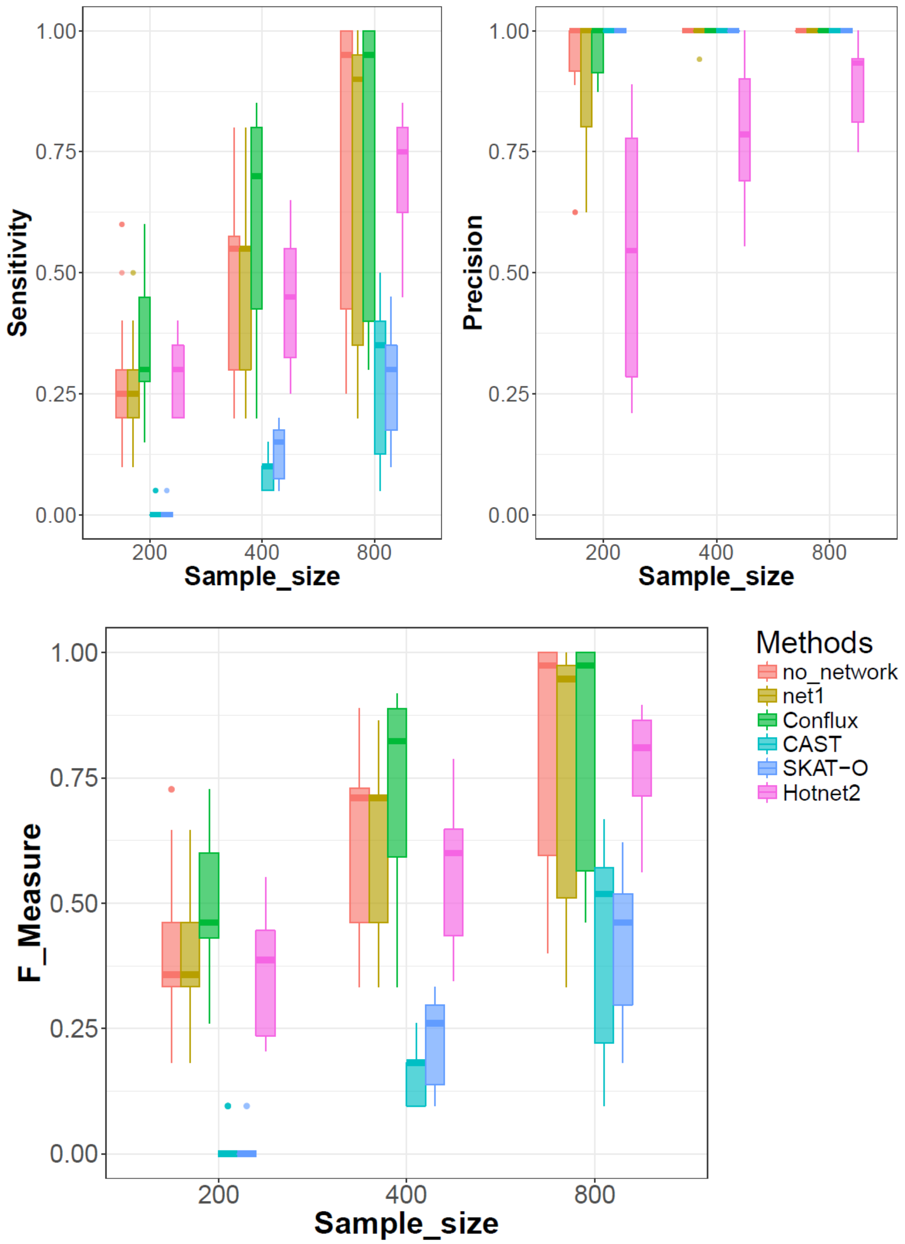

Figure 1: Sensitivity, Precision and F-Measure as a function of the sample size, evaluated for our method (Conflux) respectively using no network information (no-network), only the direct neighbours for each gene (net1), and the second order neighbourhood around each gene (Conflux). We also show the performance of SKAT-O, the burden test CAST and Hotnet2. The sample size is varied from 200 to 800 on the x-axis (the sample size is equal to the number of cases plus the number of controls).

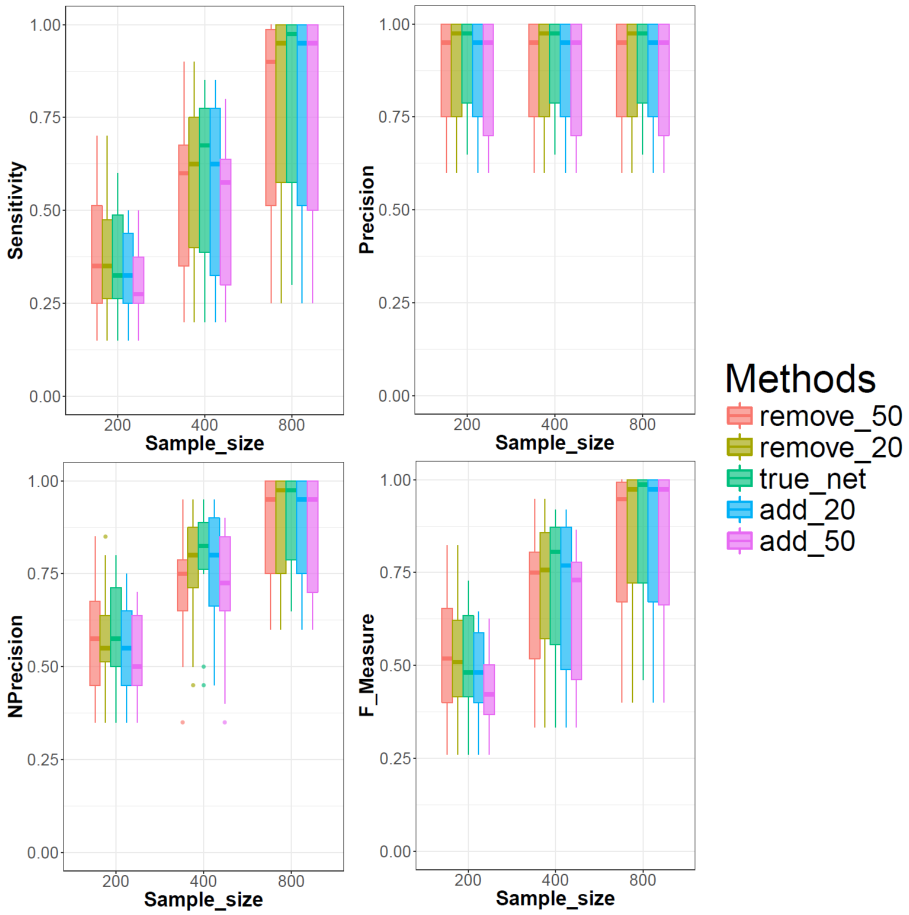

Figure 2: Sensitivity, Precision, N-Precision (ranking performance) and F-Measure as a function of the sample size, evaluated for our method (Conflux) for different perturbations on the PPI network. We show the performance using the original network and after removing or adding random edges to the network. The sample size is varied from 200 to 800 on the x-axis (the sample size is equal to the number of cases plus the number of controls).

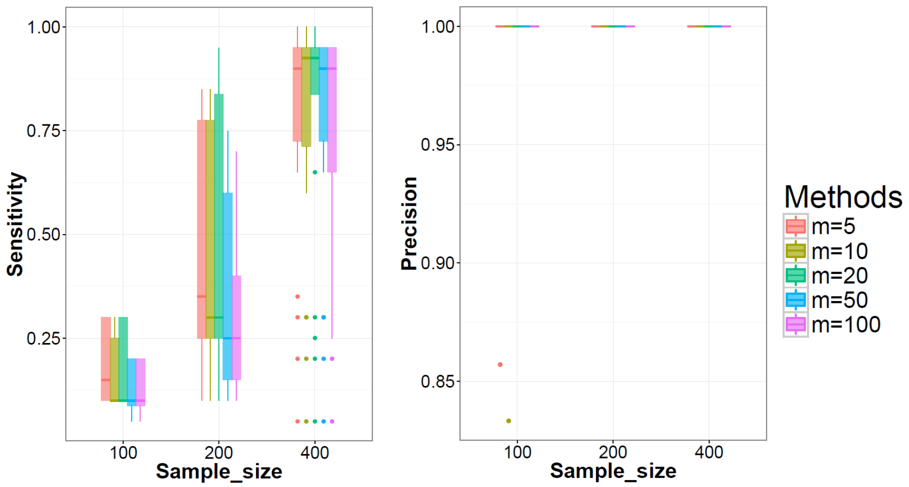

Figure 3: Sensitivity and Precision as a function of the sample size, evaluated for our method (Conflux) for different values of the Tau parameter. m is tau multiplied by the total number of genes. The sample size is varied from 200 to 800 on the x-axis (the sample size is equal to the number of cases plus the number of controls). We simulated 20 causal genes in each experiment (sampled from random neighbourhoods similarly to what we presented in the main paper). A bar represents 20 experiments.

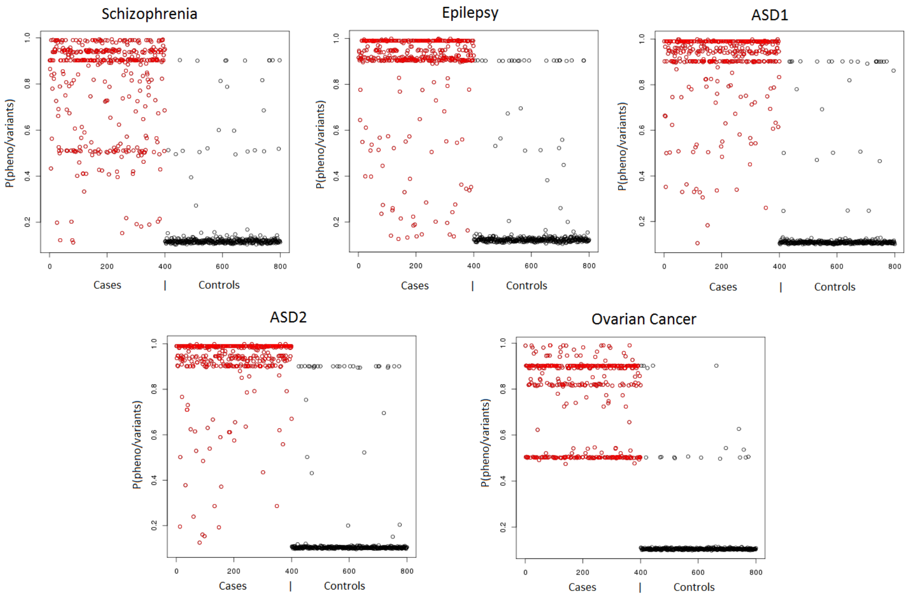

Figure 4: Predicted probabilities of being a case for all 800 individuals in each dataset. 400 cases (red, left) and 400 controls (black, right) in each figure.
